# Taste Changes and Salivary Flow Rate Disparities in Premenopausal and Postmenopausal Women: Exploring the Zinc Connection

**DOI:** 10.7759/cureus.62538

**Published:** 2024-06-17

**Authors:** Divya Harika Pedada, Komali Garlapati, Raj Kumar Badam, Poojitha Gone, Ayesha Aiman, Hanmandla Rajani, Sri Sahithya Kataram, Mansi Kulkarni, Anulatha Manne, Manogyna Bontha

**Affiliations:** 1 Oral Medicine and Radiology, Panineeya Institute of Dental Sciences and Research Centre, Hyderabad, IND; 2 Dentistry, Kamineni Institute of Medical Sciences, Hyderabad, IND

**Keywords:** menopausal women, oral health, salivary flow rate, taste perception, zinc status

## Abstract

Introduction: Menopause triggers hormonal changes that can manifest in oral symptoms like dysgeusia, xerostomia, and burning sensations, substantially impacting daily life, including chewing, swallowing, and taste perception. Menopausal women are particularly susceptible to nutritional fluctuations, including variations in zinc levels, which are believed to be linked to taste perception. Taste alterations can render food unappetizing, leading to malnutrition and diminished quality of life. The study aims to assess taste alterations, salivary flow rate, and zinc levels in premenopausal and postmenopausal women, investigating the correlation between these factors.

Materials and methods: This research involved 30 premenopausal and 30 postmenopausal women randomly selected from outpatients at the Department of Oral Medicine and Radiology, Panineeya Institute of Dental Sciences and Research Centre, Hyderabad, India. Saliva samples were collected, unstimulated salivary flow rates were measured, taste perception was evaluated using a whole mouth threshold taste test, and serum zinc levels were assessed.

Results: The study revealed that a significantly higher percentage of postmenopausal women could not identify sucrose taste at concentrations 1 (76.7%, p = 0.017) and concentrations 2 (56.7%, p = 0.007) compared to premenopausal women (43.3%, 20%, respectively). A statistically significant number of postmenopausal women also couldn't identify the bitter taste at concentration 1 (43.3%, p=0.047) compared to premenopausal women. No significant difference in taste perception of salt and sour was observed between both groups at all tested concentrations. In both groups, mean taste perception rankings were similar, with salt being most perceived, followed by sour bitter, and at least with sucrose. Salivary flow rates and zinc levels did not significantly differ between premenopausal and postmenopausal women. The correlation between zinc levels and taste perception was weak and non-significant, indicating that zinc levels were not significant predictors of taste perception in either group.

Conclusion:Postmenopausal women exhibited reduced perceptions of sucrose and quinine hydrochloride, potentially impacting eating habits, while taste perception of sodium chloride and citric acid remained relatively consistent. Salivary flow rates and zinc levels were within the normal range for postmenopausal women up to 60 years of age included in the study. The study demonstrated that zinc levels did not significantly influence perception among postmenopausal women, suggesting that taste impairment is a multifactorial phenomenon.

## Introduction

Women experience unique health challenges throughout their lives due to the profound physiological changes driven by hormonal fluctuations. Menopause is a natural event typically occurring between the ages of 45 and 55, marking the end of a woman’s reproductive years, signaling the cessation of menstrual cycles [1]. It exerts significant and multifaceted impacts on the oral cavity, extending beyond the well-documented systemic effects on women’s health. Postmenopausal women commonly complain of a range of oral discomforts, including dry mouth, increased viscosity of saliva, burning sensation, altered taste perception, various mucosal disorders such as lichen planus, benign mucosal pemphigoid, and Sjogren's syndrome. While it is essential to acknowledge that these oral changes may potentially be related to systemic diseases or medication side effects, the association between menopause and oral health alteration cannot be dismissed [2]. Among the numerous facets of oral health affected during the transition, taste perception, salivary flow rate, and zinc status are often overlooked but can play a significant role in the overall well-being of menopausal women.

Taste changes and alterations in salivary flow affect dietary habits, nutrition, and overall quality of life. According to available data, 27.1% of postmenopausal women report experiencing dry mouth, while 3.6% report altered taste [2]. The intricate connection between these two phenomena becomes readily apparent, where each influences others in various ways. Saliva acts as a solvent that dissolves and transports taste molecules and aids in interacting taste molecules with taste receptors. Strong, enjoyable taste perception triggers salivary flow rate and salivary reflexes. Zinc, an essential micronutrient, is at the epicenter of these two physiological processes. It is a cofactor for many enzymes central to taste perception and vital in maintaining salivary gland function [3]. Hence, this study was planned to assess taste alterations, salivary flow rate, and serum zinc levels in premenopausal and postmenopausal women and to compare the interrelationship between salivary flow rate, taste alterations, and zinc status in both groups.

## Materials and methods

Study location and participant selection

The study was conducted at the Department of Oral Medicine and Radiology in Panineeya Mahavidyalaya Institute of Dental Sciences and Research Centre, Hyderabad, India, from 2020 to 2022. The study was approved by the Institutional Ethical Committee of Panineeya Mahavidyalaya Institute of Dental Sciences and Research Center (approval number: PMVIDS&RC/IEC/OMR/DN/356-20). The study adhered to the Declaration of Helsinki and the World Medical Association's code of ethics. All participants gave consent, and confidentiality was maintained.

Female patients who met the criteria were included in the research through a simple random sampling method. A total of 60 participants, 30 healthy premenopausal and 30 healthy postmenopausal women aged 30-60 years, with informed consent, were selected for the study. Inclusion criteria comprised premenopausal healthy women with regular menstrual cycles, postmenopausal women, and individuals without deleterious habits such as smoking, tobacco chewing, or alcoholism within the age range of 30-60 years. Exclusion criteria involved individuals with systemic diseases, oral mucosal diseases, those undergoing hormone replacement therapy (HRT), and those on drugs that affect salivary flow and taste. Additionally, individuals with salivary gland disorders, individuals with gustatory dysfunction due to infections, those with psychological disorders, pregnant individuals, patients with dentures, and those who did not reach menopause physiologically were excluded.

Data collection

A detailed case history was recorded, with each subject comfortably seated on the dental chair and asked for the required information relevant to the study.

Estimation Of Salivary Flow Rate

**Figure 1:**
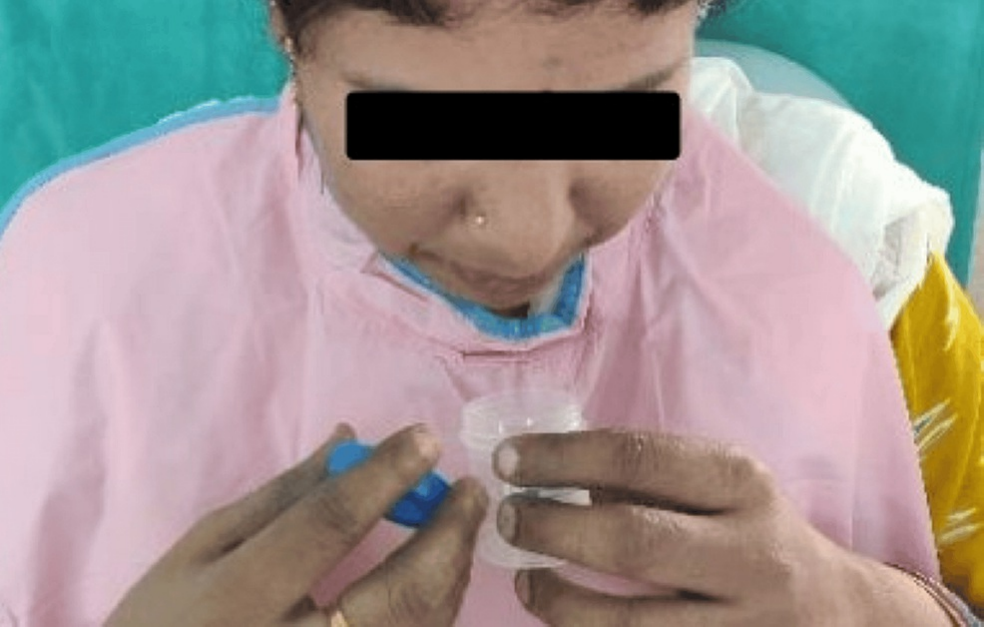
Unstimulated saliva collection

The salivary flow rate was determined by considering the diurnal quantitative salivary secretions. All measurements were recorded in the late morning in a quiet, distraction-free room. Unstimulated whole saliva was collected. Prior to collection, participants were asked to rinse their mouths with water and swallow any existing saliva present in their mouths. They were then instructed to allow new saliva to accumulate in their mouths and to expectorate it into a sterile graduated receptacle every 60 seconds for a period of five minutes (Figure 1). Following collection, the quantity of the saliva was measured with a micropipette.

Taste Evaluation

**Figure 2:**
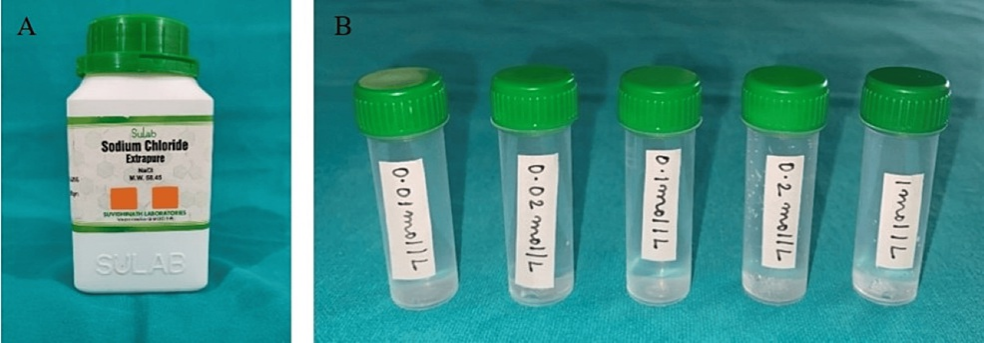
(A) Sodium chloride container; (B) Sodium chloride solutions at five concentration levels (in ½ log steps) (0.01 mol/l, 0.02 mol/l, 0.1 mol/l, 0.2 mol/l, 1 mol/l) mol/l: moles per liter

**Figure 3:**
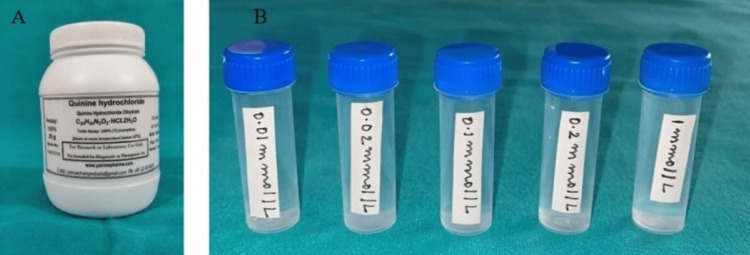
(A) Quinine hydrochloride container; (B) Quinine hydrochloride solutions at five concentration levels (in ½ log steps) (0.01 mmol/l, 0.02 mmol/l, 0.1 mmol/l, 0.2 mmol/l, 1 mmol/l) mol/l: moles per liter; mmol/l: millimoles per liter

**Figure 4:**
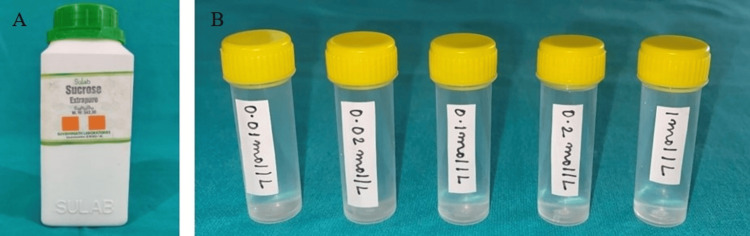
(A) Sucrose container; (B) Sucrose solutions at five concentration levels (in ½ log steps) (0.01 mol/l, 0.02 mol/l, 0.1 mol/l, 0.2 mol/l, 1 mol/l) mol/l: moles per liter

**Figure 5:**
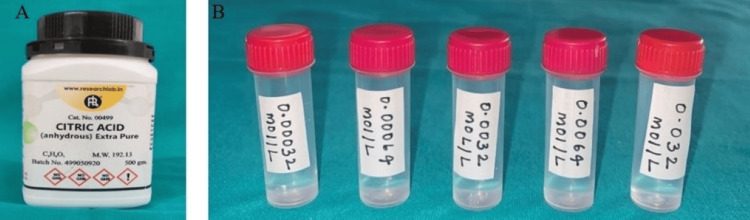
(A) Citric acid container; (B) Citric acid solutions at five concentration levels (in ½ log steps) (0.32 mmol/l, 0.64 mmol/l, 0.0032 mol/l, 0.0064 mol/l, 0.032 mol/l) mol/l: moles per liter; mmol/l: millimoles per liter

**Figure 6:**
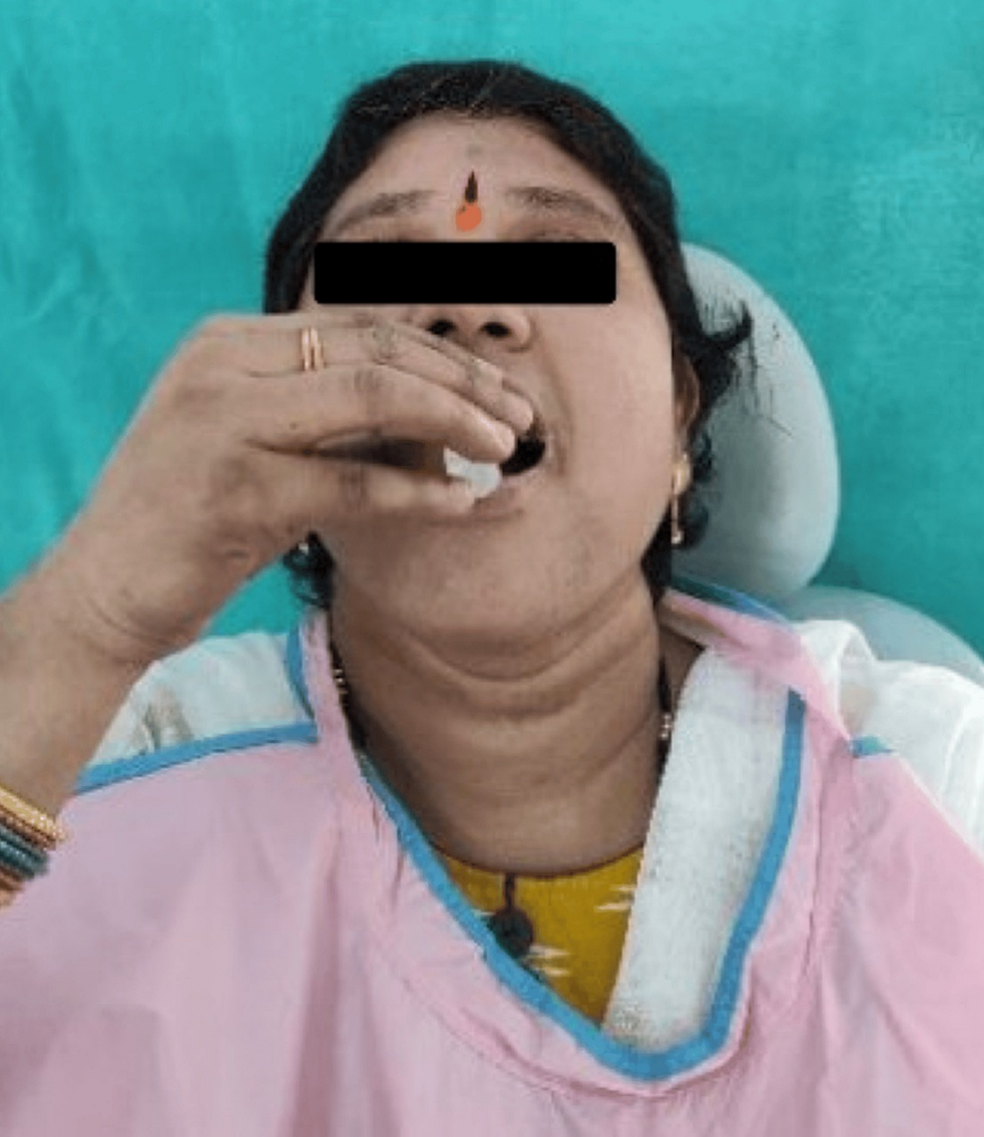
Participant tasting taste solution

A whole mouth threshold taste test was conducted, involving five concentration levels (in ½ log steps) of sodium chloride (0.01mol/l-1.0 mol/l) (Figure 2), quinine hydrochloride (0.01mmol/l-1.0 mmol/l) (Figure 3), sucrose (0.01mol/l-1.0mol/l) (Figure 4), and citric acid (0.32mmol/l-0.032 mol/l) (Figure 5) in color-coded containers. Before tasting, participants were asked to rinse their mouths with plain water. They were instructed to pour 5 ml of one of the samples into their mouth and hold it there for five seconds before spitting it out (Figure 6). The participants were asked to identify the solution. After sampling each solution, participants were instructed to rinse their mouths with water twice and wait 30 seconds before proceeding to the next sample. All four samples are tasted and evaluated similarly.

Zinc Evaluation

Whole blood samples were collected in a plain vacutainer (red cap) for the estimation of serum zinc levels by Photometric analysis using a spectrophotometer analyzer (ADVIA® 2400 Clinical Chemistry System; Siemens AG, Munich, Germany) was employed for quantitative assessment of serum zinc levels (Nitro-PAPS)

Statistical analysis

The statistical analysis was done using IBM SPSS Statistics for Windows, Version 22.0 (Released 2013; IBM Corp., Armonk, New York, United States). The Chi-square test was done to compare taste perception of individual tastes at different concentrations in healthy premenopausal and healthy postmenopausal women. Mann-Whitney U test compared mean taste perception, salivary flow rates, and zinc levels in pre and post-menopausal women. The Spearman correlation test determined the association between taste perception and zinc. Linear regression analysis is used to predict the strength of the association between zinc and taste perception.

## Results

Taste perception in premenopausal and postmenopausal women

Evaluation of Individual Taste Perception at Different Concentrations

A statistically significant number of postmenopausal women could not identify sucrose taste at concentrations 1 (P=0.017) and 2 (P=0.007) compared to premenopausal women. No significant difference was observed between premenopausal and postmenopausal women at concentration 3. All individuals could identify sucrose taste at concentrations 4 and 5 (Table 1). No significant difference in the taste perception of salt was observed between premenopausal and postmenopausal women at all concentrations tested. A comparably lesser percentage of postmenopausal women could perceive the salt taste at concentrations 1 (P= 0.254) and 2 (P=1.000) in comparison to premenopausal women. All individuals were able to identify salt taste at concentrations 3, 4, and 5 (Table 2). No significant difference in sour taste perception was observed between premenopausal and postmenopausal women at all concentrations tested. Only a small percentage of postmenopausal women could not perceive the sour taste at concentration 1 (P= 1.000) in comparison to premenopausal women. All individuals could identify taste at concentrations 2, 3, 4, and 5 (Table 3). A statistically significant number of postmenopausal women could not identify bitter taste at concentration 1 (P=0.047) compared to premenopausal women. At concentrations 2 and 3, no significant difference was observed between premenopausal and postmenopausal women. All individuals could identify bitter taste at concentrations 4 and 5 (Table 4).

**Table 1:** Sucrose perception in the two groups at different concentrations The analysis is done using the chi-square test; p≤0.05 considered statistically significant The asterisk (*) indicates statistically significant findings

Taste perception		Premenopausal women (N=30)		Postmenopausal women (N=30)		p-value
		No	Yes	No	Yes	
		n (%)	n (%)	n (%)	n (%)	
Sucrose	Concentration 1	13 (43.3)	17 (56.7)	23 (76.7)	7 (23.3)	0.017*
	Concentration 2	6 (20)	24 (80)	17 (56.7)	13 (43.3)	0.007*
	Concentration 3	1 (3.3)	29 (96.7)	4 (13.3)	26 (86.7)	0.353
	Concentration 4	0 (0)	30 (100)	0 (0)	30 (100)	-
	Concentration 5	0 (0)	30 (100)	0 (0)	30 (100)	-

**Table 2:** Salt perception in the two groups at different concentrations The analysis is done using the chi-square test; p≤0.05 considered statistically significant

Taste perception		Premenopausal women (N=30)		Postmenopausal women (N=30)		p-value
		No	Yes	No	Yes	
		n (%)	n (%)	n (%)	n (%)	
Salt	Concentration 1	2 (6.7)	28 (93.3)	6 (20)	24 (80)	0.254
	Concentration 2	0 (0)	30 (100)	1 (3.3)	29 (96.7)	1.000
	Concentration 3	0 (0)	30 (100)	0 (0)	30 (100)	-
	Concentration 4	0 (0)	30 (100)	0 (0)	30 (100)	-
	Concentration 5	0 (0)	30 (100)	0 (0)	30 (100)	-

**Table 3:** Sour perception in the two groups at different concentrations The analysis is done using the chi-square test; p≤0.05 considered statistically significant

Taste perception		Premenopausal women (N=30)		Postmenopausal women (N=30)		p-value
		No	Yes	No	Yes	
		n (%)	n (%)	n (%)	n (%)	
Sour	Concentration 1	0 (0)	30 (100)	1 (3.3)	29 (96.7)	1.000
	Concentration 2	0 (0)	30 (100)	0 (0)	30 (100)	-
	Concentration 3	0 (0)	30 (100)	0 (0)	30 (100)	-
	Concentration 4	0 (0)	30 (100)	0 (0)	30 (100)	-
	Concentration 5	0 (0)	30 (100)	0 (0)	30 (100)	-

**Table 4:** Bitter perception in the two groups at different concentrations The analysis is done using the chi-square test; p≤0.05 considered statistically significant The asterisk (*) indicates statistically significant findings

Taste perception		Premenopausal women (N=30)		Postmenopausal women (N=30)		p-value
		No	Yes	No	Yes	
		n (%)	n (%)	n (%)	n (%)	
Bitter	Concentration 1	5 (16.7)	25 (83.3)	13 (43.3)	17 (56.7)	0.047*
	Concentration 2	2 (6.7)	28 (93.3)	3 (10)	27 (90)	1.000
	Concentration 3	0 (0)	30 (100)	1 (3.3)	29 (96.7)	1.000
	Concentration 4	0 (0)	30 (100)	0 (0)	30 (100)	-
	Concentration 5	0 (0)	30 (100)	0 (0)	30 (100)	-

Evaluation of Mean Taste Perception In Premenopausal Women, Post-menopausal Women, and Overall Female Sample

**Figure 7:**
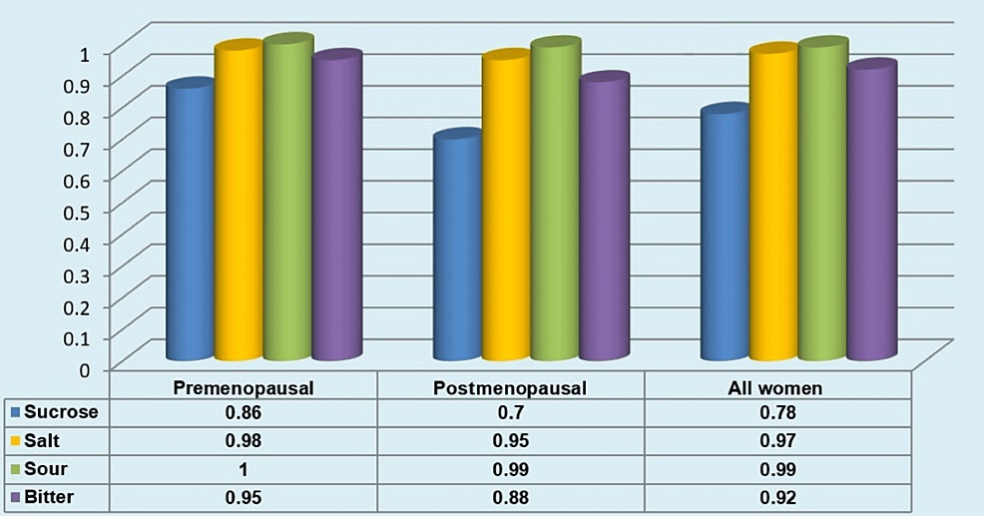
Mean taste perception in premenopausal, postmenopausal, and in overall female population

**Figure 8:**
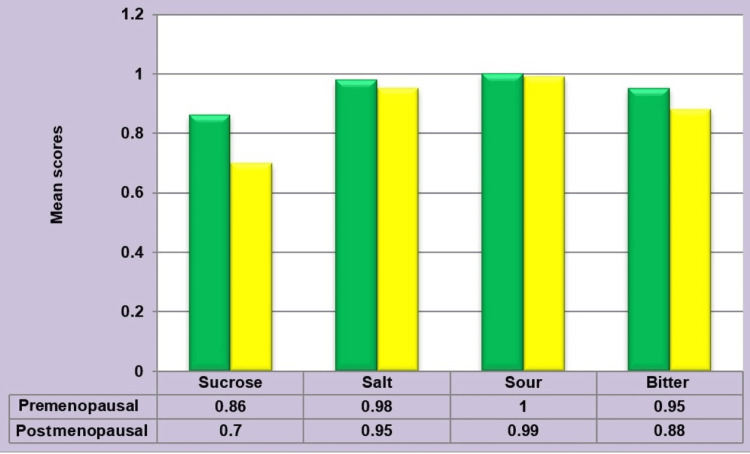
Comparison of mean taste perception between the two groups The Mann-Whitney U test was employed.

Among premenopausal women, postmenopausal women, and the overall sample, the mean taste perception was highest for salt, followed by sour, bitter, and lowest for sucrose (Figure 7). The mean perception of sucrose, salt, sour, and bitter was comparably lower in postmenopausal women (0.70±0.20, 0.95±0.10, 0.99±0.03, 0.88±0.15, respectively) than in premenopausal women (0.86 ±0.176, 0.98±0.05074, 1.00±0.00, 0.95±0.11, respectively) (Figure 8). 

Salivary flow rate in premenopausal and postmenopausal women

**Figure 9:**
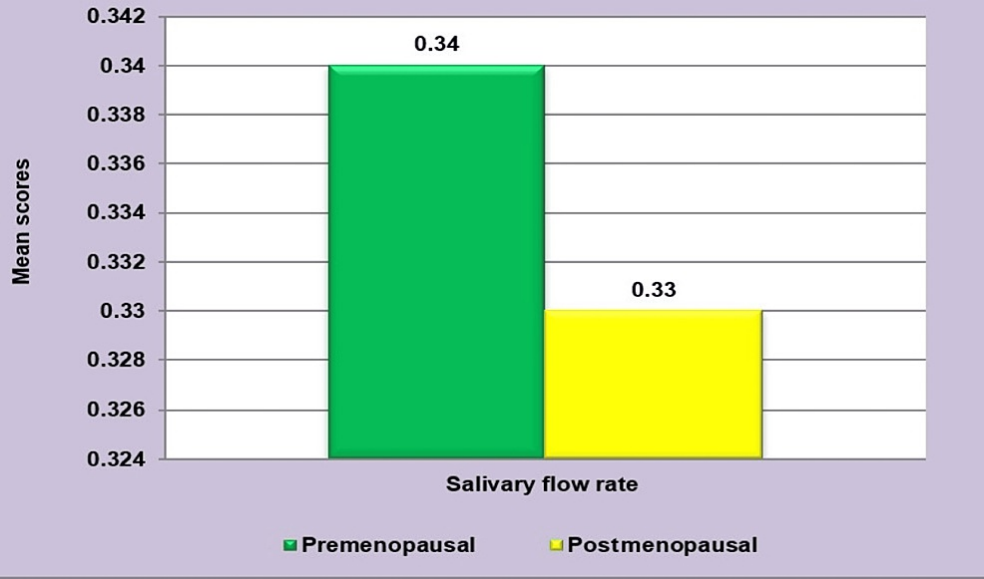
Mean comparison of salivary flow rate between the two groups The Mann-Whitney U test was employed.

It was observed that mean salivary flow rate levels in postmenopausal women were slightly lower than in premenopausal women, but there was no statistically significant difference (P =0.413) (Figure 9).

Zinc and taste perception

**Figure 10:**
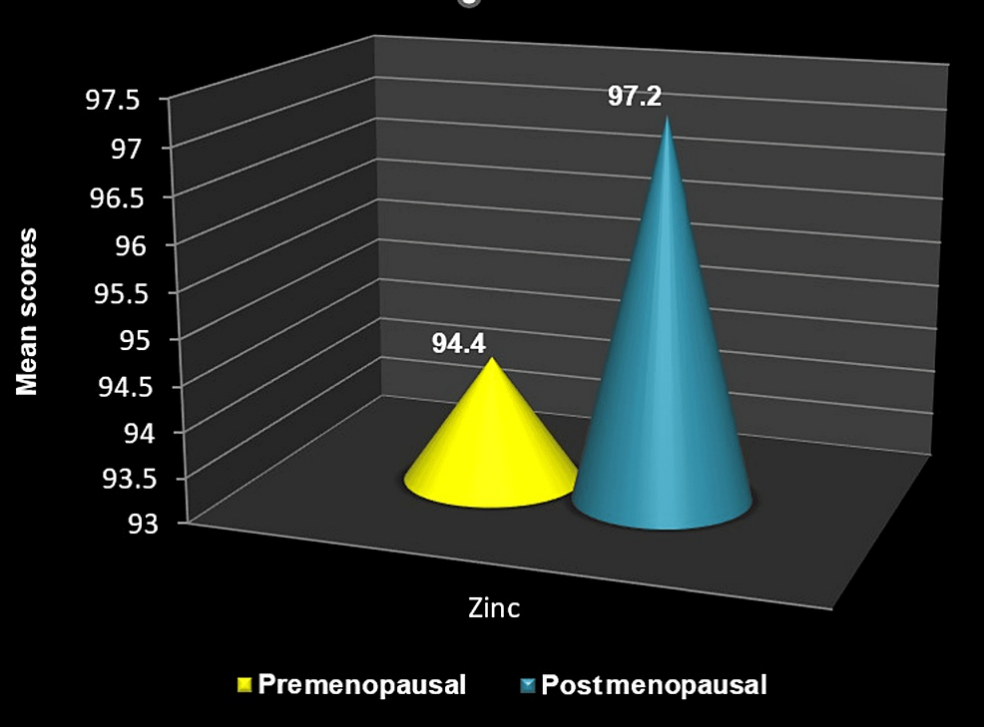
Comparison of mean zinc levels between premenopausal and postmenopausal women The Mann-Whitney U test was employed.

The mean zinc levels in postmenopausal women are slightly higher than in premenopausal women, but the difference was statistically non-significant (P = 0.912) (Figure 10). The relationship between taste perception and zinc levels was analyzed in premenopausal and postmenopausal women. Premenopausal women who could perceive the taste of sucrose, salt, and bitter substances generally showed slightly higher levels of zinc than those who could not (P= 0.536, 0.860, 0.733, respectively). Similar observations were made for postmenopausal women; those who could perceive the taste of sour, salt, and bitter substances had slightly higher zinc levels with no statistical significance. (P = 0.848, 0.432, 0.157) However, postmenopausal women who could perceive the taste of sucrose showed slightly lower levels of zinc than the women who could not perceive the sucrose taste (P=0.848). The Spearman correlation test concluded that the correlation between all the taste perceptions examined and zinc levels is weak and nonsignificant (Table 5). Linear regression analysis was used to predict the strength of the association between zinc levels and taste perception. It was observed that zinc levels are not a significant predictor in the perception of any of the tastes examined among both pre and post-menopausal women and the overall sample (Table 6).

**Table 5:** Correlation of zinc levels with taste perception in premenopausal, postmenopausal, and overall female population The Spearman correlation test was employed; p≤0.05 considered statistically significant

		Premenopausal women	Postmenopausal Women	Overall sample
Sucrose	R value	0.108	-0.167	-0.017
	P value	0.570	0.378	0.898
Salt	R-value	0.015	0.283	0.175
	P value	0.935	0.130	0.181
Sour	R-value	-	0.311	0.222
	P value	-	0.094	0.089
Bitter	R-value	-0.206	0.089	-0.037
	P value	0.275	0.641	0.776

**Table 6:** Strength of association between zinc levels and taste perception in premenopausal, postmenopausal, and overall female population Linear regression analysis was employed.

		Unstandardized coefficients		Standardized coefficients	t	Sig.
		B	Std. Error	Beta		
Premenopausal women	sucrose	11.463	25.791	0.095	0.444	0.660
	salt	14.080	94.040	0.034	0.150	0.882
	sour	-	--	-	-	-
	bitter	-19.449	39.334	-0.104	-0.494	0.625
Postmenopausal women	sucrose	-66.688	37.905	-0.352	-1.759	0.091
	salt	108.428	93.941	0.286	1.154	0.259
	sour	-160.839	311.325	-0.154	-0.517	0.610
	bitter	43.914	57.676	0.178	0.761	0.454
All samples	sucrose	-33.284	21.377	-0.222	-1.557	0.125
	salt	80.169	65.234	0.211	1.229	0.224
	sour	25.242	217.305	0.021	0.116	0.908
	bitter	9.637	34.666	0.044	0.278	0.782

## Discussion

Oral health often gets overlooked despite its significant impact on overall well-being. While many oral sensory complaints are traditionally associated with oral diseases, it is crucial to understand that certain manifestations may be linked to systemic disease. Numerous authors have proposed a potential relationship between hormonal imbalances experienced during menopause and the spectrum of oral sensory complaints.

Clinical observations by Wardrop et al. revealed that about two-thirds of menopausal women with oral discomfort who did not show oral clinical signs reported relief from this symptom following hormonal replacement therapy [4]. Tarkkila et al. have suggested that the occurrence of painful mouth and dry mouth seemed to be associated with climacteric symptoms in general, but the use of hormonal replacement therapy did not prevent the oral symptoms [5]. Both studies suggested that since the oral mucosa contains estrogen receptors, fluctuations in hormone levels may have an impact on the oral cavity [4,5]. However, not all studies point exclusively to hormonal influences. Bercovici et al. suggested that local irritants could significantly cause oral discomfort in postmenopausal women [6]. According to Delilbasi et al., oral discomfort in postmenopausal women may have a number of diverse origins, some of which might not be directly related to hormonal changes [7]. An association between oral discomfort and psychological symptoms in menopausal women was also reported by Wardrop et al. [4].

Taste perception in premenopausal and postmenopausal groups

One of the first human senses, taste, plays a crucial part in an organism's ability to survive and eat. Complaining about taste is seen in two ways: complaining about reduction or loss of the sense of taste (hypogeusia or ageusia) or continuous unpleasant taste in the mouth (phantogeusia), often related to the change in the quality of flavor (dysgeusia) [3].

The participants in the current study showed a significant reduction in the intensity of taste perception for both sweet and bitter taste in postmenopausal women compared to premenopausal women. Conversely, there was no difference in the intensity of taste perception of sodium chloride and citric acid between the two groups, concluding that there is no decline in sensitivity to salty and sour taste, respectively, in postmenopausal women. Also, out of 13 individuals who had decreased sensitivity to bitter taste, 12 also had decreased sensitivity to sucrose taste. However, out of 23 individuals who had decreased sensitivity to sucrose taste, only 12 had decreased sensitivity to bitter taste. This observation indicates that decreased sensitivity to bitter taste is more likely to be associated with decreased sensitivity to sweet taste, whereas the reverse is less frequently observed. i.e., bitter taste sensitivity appears to have a stronger influence on sweet taste sensitivity than vice versa in this particular group of participants.

Previous studies on the effect of menopause on taste perception have revealed diverse results. Delilbasi et al. revealed a substantial decrease in taste perception of sweet (sucrose) and its palatal sensitivity in postmenopausal women [7], similar to the current study. However, bitter taste sensitivities did not differ significantly between postmenopausal women and men. Dangore-Khasbage et al. also noted significantly reduced intensity of taste perception for sucrose in postmenopausal women compared to age-matched males but perceived no significant differences in salty, sour, and bitter tastes [8]. Saluja et al. stated that there was a significant difference in mean total taste intensity ratings for sucrose between the postmenopausal group and the control group but was not significant when compared between the premenstruating, pregnant, and control groups [9]. Kalantari et al. concluded that the strength of the sweet taste perception was significantly lower among women after menopause; however, there was no significant difference between the perception of other tastes among postmenopausal women and men of the same age [10]. Agarwal et al. concluded that there was significantly reduced sweet intensity and pH in postmenopausal women than in menstruating women [11]. The current study aligns with these findings, showing reduced sweet taste sensitivity in postmenopausal women. However, additionally, it also shows a significantly reduced intensity of taste perception for the bitter taste in postmenopausal women.

Numerous studies have explored potential causes of dysgeusia in women, revealing a multifaceted origin with the interplay of biological and psychological systems. Formaker and Frank’s study revealed a consistent reduction in sucrose perception along with an alteration in the perception of sodium chloride [12]. They proposed that these changes in neural and behavioral taste function may be attributed to the activation of pain pathways. Bartoshuk et al.'s findings indicated a correlation between higher body mass index (BMI) and diminished perceived sweetness, and reduced sweetness perception coincided with a greater preference for fatty foods over sweet ones [13]. This implies a potential connection between the decline in sucrose sensitivity in postmenopausal women and dietary modifications. Few studies have highlighted age-related decline in taste function. Cowart et al. found that individuals over 65 years of age reported phantogeusia and a reduction of taste significantly more than young and middle-aged patients [14]. In the present study, to rule out the influence of aging changes, a female population of only 30-60 years was considered.

Saluja et al. reported that, during a woman’s developmental journey from birth to menopause, taste preferences change [9]. Postmenopausal women, in particular, rated sweet as the most pleasant taste. Kuga et al.’s research highlighted a preference for sour taste in pregnant women [15]. Verma et al., on the other hand, observed cyclic variation in salt preference in females throughout different phases of the menstrual cycle [16]. This preference may be attributed to the body's need for electrolytes during pregnancy and to support both the mother's and the developing child’s nutritional needs. Delilbasi et al. pointed out that factors beyond aging, such as smoking habits, dietary choices, and dentition status, can contribute to the gradual loss of taste [7]. In the current study, the decrease in taste sensitivity among postmenopausal women was not associated with any above-specified event. Ahne et al. highlighted a common confusion between salty and sour tastes due to slight tongue-stinging characteristics [17], a confusion not observed in the present study.

In the present study, a whole mouth taste test was employed, which yielded statistically significant results related to sweet and bitter tastes between study and control groups. Although regional or spatial taste-testing systems are also employed to assess the gustatory function, they come with limitations. When using cotton swabs or small pieces of filter paper to apply tastant, there is poor diffusion, and using drops of solutions for tongue stimulation lacks precise control over the spatial or regional extent of the stimulus on the tongue’s surface. Hence, regional or spatial taste testing methods are more suitable for cases with localized taste impairment resulting from conditions like post-surgery, tumor, stroke, and viral infections. Whole-mouth tests of gustatory function are necessary to assess everyday taste experiences that are not adequately represented by regional tests [17].

Mean salivary flow rate in premenopausal and postmenopausal groups

Women may be particularly vulnerable to salivary alterations due to distinct hormonal shifts they experience. Researchers have proposed that menopause could affect the performance of salivary glands, given the presence of sex hormone receptors in these glands [18]. Several studies have investigated the relationship between dry mouth and menopause by assessing salivary function in postmenopausal women. While some authors have linked menopause to reduced salivary flow, others have been unable to detect changes in either salivary volume or composition.

In the current study, the findings indicated that there was no significant disparity in mean salivary flow rate between healthy postmenopausal (0.33+0.05) and premenopausal women (0.34±0.05) (P =0.413). Therefore, the present study suggests that the salivary flow rate can remain within the normal range until 60 years of age in postmenopausal women.

Some studies align with the present study. A longitudinal study conducted by Ship et al. reported no significant alteration in the quantity of salvia between premenopausal and postmenopausal women [19]. The hypothesis proposed in their study attributed these findings to changes from acinar destruction caused by aging processes.

In contrast to the present study, Tremblay et al. noted a distinct reduction in unstimulated saliva levels after menopause [20]. Additionally, Bhat et al. [21] and Rukmini et al. [22] demonstrated a decline in salivary flow rates post-menopause accompanied by lower salivary pH. Post-menopausal women also exhibited higher Oral Hygiene Index (OHI) and Decayed, Missing, and Filled Teeth (DMFT) values when compared to the control group. This decline in salivary pH and flow rate in postmenopausal women contributes to OHI and DMFT indices. Gill et al. reported that specific symptoms like taste disorders and dry mouth were more pronounced in postmenopausal women and were associated with lower salivary flow rates [18]. Cydejko et al. found statistically significant differences in values of salivary flow rate and lysozyme and ionized calcium concentrations between pre and postmenopausal women [23]. Mahesh et al. not only observed a marked reduction in salivary flow rate among postmenopausal women compared to menstruating women but also noted an enhancement in the flow rate in individuals undergoing HRT [24]. This study emphasizes that postmenopausal women may experience oral discomfort, while HRT has the potential to ameliorate these issues.

In the present study, although there was no significant decrease in salivary flow rate in postmenopausal women, it was observed that 15% of postmenopausal women had a decrease in salivary flow rate. Hence it is recommended that studies with a larger sample size be needed to validate the results. Research linked possible alteration of salivary secretion to systemic diseases and age changes that can have a confounding effect. Results in other studies could be influenced by the broad variation of aging within the sample [20-22].

Zinc levels in premenopausal and postmenopausal women

While reduced food intake or impaired dietary absorption typically contribute to zinc deficiency in older women, postmenopausal women may also encounter a decline in serum zinc levels. The significance of zinc in menopausal nutrition and overall health has garnered increasing attention in recent years. As a result, numerous experts have identified zinc deficiency as a noteworthy public health concern, particularly in underdeveloped regions [25].

Postmenopausal women are more susceptible to serum biochemical alterations, potential nutritional imbalances, including deficiencies in trace elements and vitamins, and hormonal shifts, increasing the risk of age-related diseases during this time. It is imperative to consider these serum changes for the early diagnosis and prevention of menopause-related diseases. In cases where levels are significantly depleted, dietary supplementation may be necessary [26].

In the present study, the mean zinc levels in postmenopausal women (97.2±38.2) were slightly higher than in premenopausal women (94.4±21.2) However, the difference was not statistically significant. Studies conducted by Gupta and Arora [27], as well as Ansar et al. [28], also did not find any significant difference in the serum zinc level between premenopausal and postmenopausal women, which is in accordance with the present study.

Contrarily, Bednarek-Tupikowska et al. observed a significantly higher concentration of serum zinc in post-menopausal women than in premenopausal women [29]. Similarly, a study on a Czech population reported that blood zinc concentration increased with age in women [30]. They have proposed that these elevated values may be attributed to increased bone resorption resulting from estrogen deficiency during the post-menopausal period. This contradicts the findings of the studies by Grochans et al. [31] and Ferdous et al. [25], who reported lower serum zinc levels in postmenopausal women, possibly due to decreased serum albumin, a known major carrier of zinc.

Based on the above findings, the status of zinc appears ambiguous. The studies in the literature related to zinc in postmenopausal women have shown conflicting results. Hence, there is a need to further investigate the status of zinc in postmenopausal women in a larger population.

Association of zinc with taste perception

Nutritional elements like zinc play a crucial role in taste perception as they are integral components of the protein involved in taste transduction. Zinc synthesizes protein gustin, which is associated with the development of taste buds. It also serves as a vital cofactor for alkaline phosphatase, a key enzyme in the taste bud membrane. Hence, Zinc is essential for the growth of taste buds and aids in preservation and repair [3]. Shatzman et al. have noted changes in taste function following alteration in salivary gustin [32]. Clinical evidence suggested that zinc deficiency is a contributing factor to taste impairment, However, the exact relationship between zinc nutrition and taste impairment remains unclear.

In the present study, It is observed that the premenopausal women who could perceive the taste of sucrose had slightly higher levels of zinc than the women who could not perceive the sucrose taste. Nonetheless, the comparison was not statistically significant at all the concentrations (P=0.536, 0.860, 0.733, respectively). Likewise, a similar finding is also observed with salt and bitter taste perception. For postmenopausal women, it was observed that those who could perceive the taste of sucrose had lower zinc levels than those who could not. Nonetheless, the comparison was not statistically significant at any concentrations. Similarly, postmenopausal women with salt, sour, and bitter taste perceptions had slightly higher zinc levels with no significant associations.

Overall, zinc levels showed a weak and nonsignificant correlation with taste perception, and they were not a significant predictor for taste perception among both premenopausal and postmenopausal women.

Case reports by Zazgomik et al. [33], and McNeil et al. [34] have shown results in accordance with the present study. They reported that oral zinc supplements may not be effective in reversing dysgeusia induced by certain medications. However, these studies may not have provided a sufficient duration of treatment to establish the efficacy of zinc supplements.

Other studies conducted by Heckmann et al. reported improvements in gustatory function and perceived taste severity with zinc supplementation [35]. Sakagami et al. also observed significant improvement in gustatory sensitivity with zinc treatments in a group of patients [36].

The multifaceted nature of taste impairment suggests that zinc level may not be the sole determinant. Other factors such as aging, hormonal changes, nutritional variations, and local influences should also be considered when exploring the causes of taste alterations.

Limitations of the study

The present study was conducted on a relatively small sample size, which may affect the generalizability of findings to a larger population. Additionally, the utilization of the whole-mouth taste testing method, while practical and efficient, is a subjective test; hence, no accurate quantification of the disorder is possible. Furthermore, it is essential to acknowledge that the current study does not demonstrate structural and functional defects of taste receptors. Although major confounding factors such as deleterious habits, age range, systemic disease oral mucosal diseases, and those undergoing hormonal replacement therapy were controlled through strict inclusion and exclusion criteria, other uncontrollable factors such as dietary habits, genetic differences, and environmental influences may impact the results.

Strengths of the study

The study design is easy to perform, requires minimum time, and causes the least discomfort to patients. In literature, zinc supplementation is prescribed for taste alteration, but the relationship between taste and zinc remains ambiguous. The present study reveals that taste alteration is not just linked to zinc but is multifactorial, wherein there is a need to investigate. The study revealed a significantly reduced intensity of bitter taste perception in postmenopausal women. Hence, further studies should be done to validate the findings.

Future Prospects

There is more to understand related to the mechanism of taste impairment alteration, inviting further development of methods to diagnose precisely. Investigation of multifactorial etiological factors related to taste perception would lead to the development of novel methods in the management of altered taste perception in postmenopausal women.

## Conclusions

Considering one-third of a woman’s life occurs after her last menstrual period, the importance of safeguarding and maintaining her oral and general health cannot be overstated. This study highlights significant alterations in taste perception, specifically a reduction in the intensity of sweet and bitter tastes in postmenopausal women compared to premenopausal women. While the salivary flow rate remains unaffected by menopause in this study, the potential for decreased flow in some individuals necessitates further research.

The weak and nonsignificant association between taste perception and zinc levels underscores the complex nature of taste impairment in menopausal women. These findings contribute to a deeper understanding of the oral challenges faced during menopause, emphasizing the need for future research and the development of management strategies for altered taste perception in postmenopausal women. This study emphasizes the need for a holistic approach to understanding women’s oral changes, considering the interplay of various factors like hormonal fluctuations, aging, psychological factors, and other nutritional elements that contribute to their overall well-being during and after menopause.
